# Engaging Turkish Immigrants in Psychotherapy: Development and Proof-of-Concept Study of a Culture-Tailored, Web-Based Intervention

**DOI:** 10.32872/cpe.5583

**Published:** 2021-12-23

**Authors:** Hanna Reich, Daniela Zürn, Ricarda Mewes

**Affiliations:** 1 Division of Clinical Psychology and Psychotherapy, Department of Psychology, Philipps-University of Marburg, Marburg, Germany; 2 Depression Research Centre of the German Depression Foundation, Department for Psychiatry, Psychosomatics and Psychotherapy, Goethe University, Frankfurt, Germany; 3 Outpatient Unit for Research, Teaching and Practice, Faculty of Psychology, University of Vienna, Vienna, Austria; Philipps-University of Marburg, Marburg, Germany

**Keywords:** engagement, motivational interviewing, psycho-education, web intervention, cultural tailoring

## Abstract

**Background:**

Culturally tailored interventions can increase the engagement and the success rate of psychotherapy in immigrant and ethnic minority patients. In this regard, the integration of the patients’ illness beliefs is a key element. Applying principles of Motivational and Ethnographic Interviewing, we developed a culture-tailored, web-based intervention to facilitate engagement of Turkish immigrant inpatients in psychotherapy.

**Method:**

The different aspects of the engagement intervention development are described and its acceptance and usefulness were tested in a proof-of-concept trial with an experimental control group design (active control condition: progressive muscle relaxation) in a sample of Turkish immigrant inpatients in Germany (N = 26). Illness perception, illness-related locus of control, and self-efficacy were assessed pre and post intervention.

**Results:**

The engagement intervention was rated better than the control condition (p = .002) and in particular, participants felt better prepared for therapy after working with it (p = .013). By working with the engagement intervention, self-efficacy increased (p = .034) and external-fatalistic control beliefs diminished (p = .021). However, half of the participants needed assistance in using the computer and web-based interventions.

**Conclusion:**

The developed intervention provides a first step towards feasible culture-tailored psychotherapeutic elements that can be integrated into routine clinical care. The first results regarding acceptance and usefulness are promising.

## Highlights

- Culturally tailored psychotherapeutic interventions are more effective than generic ones.
- We explored the use of native language, web-based interventions for ethnic minority patients.
- An engagement intervention facilitated feeling ready for psychotherapy in Turkish immigrants.
- Web-based interventions can address complex themes such as motivational factors and illness beliefs.

Prevalence rates of psychological distress and disorders are higher in many ethnic minority populations than in the general population (Aichberger et al., 2010; de Wit et al., 2008; Sariaslan et al., 2014). Psychotherapy is a well-established and effective treatment for many mental disorders, but its interventions are based in European tradition and may be difficult to embrace for ethnic minorities (Priebe et al., 2011). Reasons for less favorable outcomes might be that socioeconomic stressors that have been reported to negatively impact mental health treatment (e.g. lower education, unemployment) are common among immigrant populations in Europe (Mösko et al., 2008; Priebe et al., 2011). Meta-analytical evidence on premature discontinuation of psychotherapy showed that low education, but not ‘race’ (i.e., the proportion of *White* patients) was a predictor of dropout (Swift & Greenberg, 2012). Moreover, conventional psychotherapy may not be sufficiently specific and can be incongruent with the cultural values and worldviews of ethnic minorities (Mösko et al., 2008; Priebe et al., 2011). Unfavorable treatment expectations, different expectations about the roles of doctors/ psychotherapists and patients, and a different understanding of illness and treatment have been shown to reduce patient motivation to seek for or engage in psychotherapy (Drieschner et al., 2004; Priebe et al., 2011; Reich et al., 2015). Last but not least, even if language, per se, is not crucial for the successful delivery of culturally appropriate psychotherapy (Benish et al., 2011), the patient must at least have some understanding of what is being said within an intervention. Limited access to interpreting services has been shown to curtail immigrant health care throughout Europe (Priebe et al., 2011).

Fortunately, some of these factors can be addressed: Preparatory interventions in advance of inpatient treatment have been shown to improve knowledge and reduce tension among patients (Best et al., 2009). Meta-analytic evidence showed that culturally adapted psychotherapy is more effective than unadapted therapy (Hall et al., 2016) and that the extent of cultural adaptation of minimally guided mental health interventions had an effect on intervention efficacy (Harper Shehadeh et al., 2016). The adaptation of the ‘illness myth’ (i.e., the subjective concepts of illness) in particular was the key moderator for a superior outcome (Benish et al., 2011). Patients’ ‘illness myths’ include, among others, treatment expectations and self-efficacy beliefs that influence the motivation for psychotherapy and treatment outcome (Drieschner et al., 2004; Hagger & Orbell, 2003). Both, subjective illness concepts and self-efficacy, can be influenced by psychological interventions such as Motivational Interviewing (Miller & Rollnick, 1991; Petrie & Weinman, 2012). An integration of techniques from Motivational Interviewing (MI) and Ethnographic Interviewing (EI) has been proposed to engage patients from ethnic minorities in psychotherapy (Swartz et al., 2007). MI is a ‘directive, client-centered counseling style for eliciting behavior change by helping clients to explore and resolve ambivalence’ (Miller & Rollnick, 1991). It is effective in a broad range of behavioral problems and diseases (Rubak et al., 2005), and is particularly helpful in clients from ethnic minority groups (Lundahl et al., 2010). Complementing MI, EI focuses on the patient’s cultural background, including perceptions of the world and its nature, values, and faith (Westby, 1990). In this regard, it encourages patients to share their own ‘narrative’, the adaptation of which Benish and colleagues (2011) found to be the key to a superior outcome in culturally adapted psychotherapy.

However, there is a lack of culturally adapted, standardized interventions for immigrant patients (Mösko et al., 2008; Priebe et al., 2011). Osilla and colleagues (2012) demonstrated how to develop and deliver a culturally relevant MI intervention successfully on the web. The use of technological platforms is considered as a strategy with great potential to address major barriers to mental healthcare (Rebello et al., 2014). Given the background outlined above, we aimed to design a web-based intervention providing inpatients with information and ideas on how they could benefit from the therapies offered in inpatient treatment. The primary goal was to encourage patients to accept psychotherapy as a culturally appropriate healing practice and thereby increase motivation for psychotherapy. The present study focused on Turkish immigrant inpatients who are among the largest immigrant populations in European countries (European Commission, 2011). Turkish immigrants reported about language problems and difficulties obtaining medical information when hospitalized (Giese et al., 2013) and inpatient treatment for common mental disorders was less successful in Turkish immigrants than in non-migrants (Mösko et al., 2008). The aims of our study were twofold: A) to develop a culture-tailored, web-based intervention to facilitate treatment engagement that can be integrated into routine clinical care without major expense, and B) to conduct a proof-of-concept study, testing the acceptance and feasibility of the intervention and its effect on motivation, control beliefs, and illness representations in a randomized controlled pilot trial.

## Materials and Method

### A) Development of the Engagement Intervention

The engagement intervention was based on MI and EI techniques and developed as a web-based tool in German and Turkish language versions for the use as one session (approx. 50 minutes) within the first two weeks of inpatient treatment for common mental disorders. We chose a bi-lingual, web-based approach to bridge the gap between patients of Turkish origin with poor knowledge of German and the German healthcare system with very scant resources of Turkish-speaking therapists. The engagement and the active control intervention were drafted in German and then fully developed in both languages simultaneously through expert discussion, pilot testing and feedback with the help of five Turkish native speakers (psychotherapists, medical doctors, professional interpreters, and university students of psychology).

#### Summary of the Contents

The intervention was named *Sağlığa Doğru* (Turkish for ‘Path to Health’) and was organized into five sections following the structure of the engagement session developed by Swartz and colleagues (2007). At the beginning, a short introduction to the structure and elements of the intervention was given. The first section of Sağlığa Doğru addressed individual symptoms, illness beliefs, and social consequences of the illness. The aim for the patients was to feel accepted, understood, taken seriously regarding their individual history, and to achieve a positive general orientation about the inpatient treatment. The second section dealt with the patients’ previous treatment experiences, allowing them to specify wishes for the current treatment. The professional help offered in the hospital was introduced as support in addition to the patients’ own resources, such as the family. The patients’ own resources were thereby validated while the integration of professional mental health care into the patients’ support system was facilitated. Educational material about the concept, process, and efficacy of psychotherapy was provided in the next section. Positive outcome expectancies regarding treatment success were encouraged by providing automated feedback using previous information entered by the patients. Section four gave the patients scope to express concerns about their treatment. In addition to practical obstacles (e.g. worries about being away from family during inpatient treatment), psychological and cultural barriers that may hamper participation in the therapy were addressed (e.g. being ashamed of symptoms, being seen as ‘crazy’). Feedback was given that such concerns are quite common, and the patients were encouraged to talk about their concerns with their therapist. The aim of the final section was to strengthen the patients’ commitment to engage in treatment. After a brief summary of the previous contents, the patients were asked to write down their individual goals for the inpatient treatment as concretely as possible, and what they could do to achieve them. A structured overview of Sağlığa Doğru is given in Table 1; the script of the engagement intervention is available as Supplementary Materials.

**Table 1:** Overview of the Engagement Intervention Sağlığa Doğru

Topic of the section	Aims	Central message	Culture-tailored web-MI elements
1. My story	Reflect upon symptoms and their social consequences; learn that therapist is validating and interested in individual story	Your personal view of your illness counts, each disease history is different.	Turkish sample patient and therapist talk about symptoms and social consequences in a video. Therapist behaves in a validating and encouraging manner. Patient is asked about his/her most impairing symptom and to check areas of life in which he/she is impaired. Written feedback corresponding to the chosen areas is provided. Patient is asked to write down his/her ‘good reason’ for therapy (‘What do you want to do again after treatment?’). Examples and hints are given.
2. Treatment – what do I already know?	Reflect upon previous treatment experiences and draw conclusions for your current treatment	You can shape your therapy – say what you like and what you don’t like!	Previous treatment experiences are queried in adapted stages. Questions about personal do’s and don’ts for the current treatment based on prior experiences (personal, hearsay, positive or negative nature of experience, personal opinion about psychotherapy). Invitation to express a wish for the treatment. Examples are given; patients are encouraged to tell their practitioners about their wish.
3. Psychotherapy can help	Learn about the efficiency and effectiveness of psychotherapy; see how a psychological model can integrate mixed causal illness attributions	Psychotherapy is an efficient and effective treatment for your disease.	Written and graphic material about process and effectiveness of psychotherapy. Video sequence in which the sample patient and the therapist develop a rationale for psychotherapeutic treatment and integrate mixed causal illness attributions (genetic predisposition, family stress, punishment from God, problems dealing with emotions) into a working model for psychotherapeutic interventions. Rating of the personal relevance of causal illness attributions addressed in the video sequence.
4. Possible obstacles	Clarify and handle (expected) treatment difficulties	It is normal to have concerns about treatment – talk about them!	Rating of the importance of different practical problems associated with inpatient treatment (e.g. unfamiliar food, difficulties to comply with religious requirements in the inpatient setting). Video in which the sample therapist asks about the sample patient’s concerns regarding treatment. Rating of the importance of psychological and cultural problems associated with psychotherapy. Feedback acknowledging the concerns and stimulating courage to talk about them openly with the therapist.
5. Next steps	Commit to engage in treatment and work for individual goals	You can influence the achievement of your goals and improve your health and life.	Open-ended questions about individual goals and actions planned. Examples from the sample patient.

#### Motivational Interviewing (MI) and Ethnographic Interviewing (EI) Elements

Sağlığa Doğru was informed by principles of MI and EI. Using open-ended questions and empathic feedback, the patients were asked about their motivation for treatment, their motivation for change, and about their own health history (cf. Table 1, Sections 1, ‘My story’, and 5, ‘Next steps’). Natural resistance to change was integrated into the intervention by actively addressing possible barriers and concerns of the patient without judgment (see Section 4, ‘Possible obstacles’). Instead, the patients’ concerns were validated by written feedback and they were encouraged to actively talk about these concerns with their therapist. This should facilitate redirection of resistance into an active client behavior in actual therapy sessions. A further goal informed by principles of MI was patient empowerment that constituted a particular aim of Sections 2 and 3 of the intervention. Knowledge about the treatment offered and an evaluation of previous treatment experiences were stimulated, as those formed the basis for informed decision making.

Principles of EI helped us to focus on the cultural background of Turkish immigrants living in Germany, especially their values and faith. We addressed typical values with video sequences of a male Turkish sample patient who talked openly about some issues prevalent in Turkish immigrants (e.g. high relevance of religious beliefs and ‘punishment from God’ as a causal illness attribution). After watching the video, the participants were asked to rate how relevant the respective attributions or concerns were to them (see Sections 3 and 4, Table 1). Encouragement to tell one’s own individual story and to actively talk about one’s own illness beliefs was given at various points throughout the intervention.

#### Culturally Adapted Elements

In order to plan and evaluate the cultural adaptations, we used the parameters suggested by Hinton and Jalal (2014) to create culturally sensitive CBT interventions, i.e., identifying the cultural group, culturally appropriate framing of CBT techniques, identifying and addressing key stressors, and incorporating key local sources of recovery and resilience. Sağlığa Doğru was culturally adapted in terms of its surface structure, e.g., the use of the native language and an ethnically matched therapist, as well as its deep structure, involving the incorporation of cultural ideas, beliefs, and values (Heim & Kohrt, 2019). Surface structure adaptations included the Turkish name Sağlığa Doğru that was used in all presentations and materials (also the German ones). Moreover, we provided a complete Turkish language version, for which idiomatic expressions and German standard terms were carefully translated. In addition, names and identities of sample patient and therapist were informed by Turkish immigrants living in Germany. For instance, a high relevance of the family and religion were taken into account. Comprehensibility for persons with low literacy was also an important goal, as many Turkish immigrants in Germany had a poor educational background. Therefore, as much information as possible was delivered using video, audio, or graphics, and sentences were kept short and grammatically simple.

Deep structure adaptations were made regarding the ingredients of psychotherapy that make it a culturally accepted ‘healing practice’: A trusting relationship between patient and therapist was modeled in video sequences by a female therapist and a male sample patient both originating from Turkey, aimed to help the patient to identify with the intervention and its contents. The therapist embedded in the program gave meaningful feedback and comprehensive information in order to foster the image of a capable ‘healer’. A common rationale for illness was developed by way of example in a video session, in which we integrated a broad variety of causal illness attributions that have been shown to be culturally relevant (Minas et al., 2007; Reich et al., 2015). To strengthen confidence in the effectiveness of psychotherapy, general information was provided in conjunction with a case vignette as an example of a patient with a Turkish migration background who got better following psychotherapy.

#### Active Control Condition

The active control intervention consisted of an applied progressive muscle relaxation (PMR) with a duration of approx. half an hour (see Table 3). The structure of the PMR was harmonized with Sağlığa Doğru and offered through a web-based platform with the same content management system. The design was interactive and patients were addressed directly. In videos, the same sample patient as in Sağlığa Doğru gave illustrative information and examples and reported on his experience with the relaxation process. After introducing the content and structure of the intervention, the purpose and principles of the muscle and breathing relaxation were explained in the first section. In addition, the participant could select answers to related questions regarding the relaxation technique. In section two, ‘My muscle and breathing relaxation’, the participant was given the opportunity to participate in a 15-minute PMR audio relaxation session with specific instructions. Then, the participants were asked about their positive and negative experiences with the relaxation, with the sample patient providing example answers. The program concluded with further information and suggestions on how to transfer the relaxation exercise to everyday life.

### B) Proof-Of-Concept Study

#### Participants and Setting

The institutional review board of the Department of Psychology, Marburg University, Germany, gave ethics approval to the study protocol. All participants provided written informed consent.

**Figure 1:**
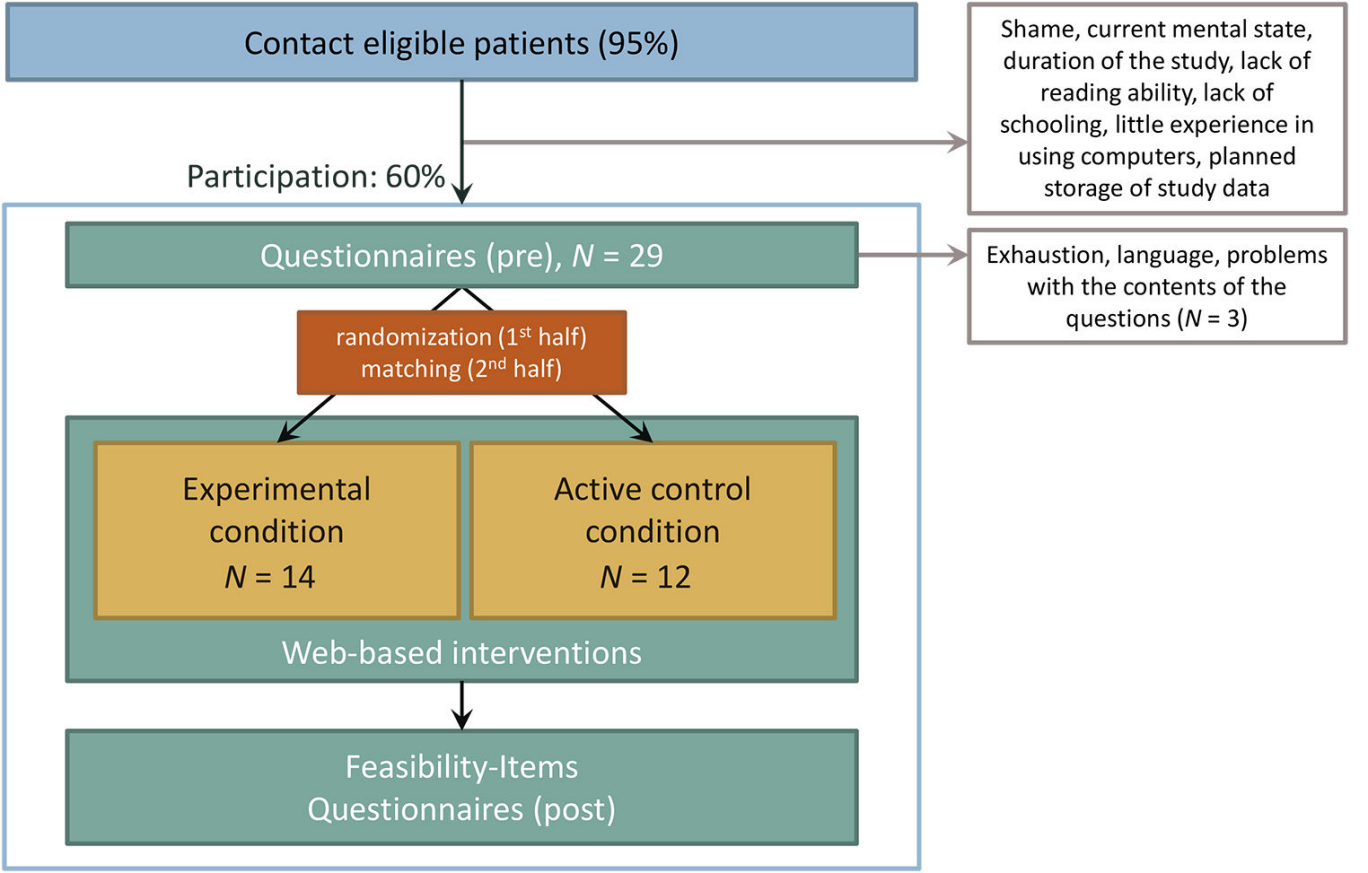
Study Design and Flow Chart

The study was based on an experimental control group design (see Figure 1) to test the feasibility and usefulness of the culture-tailored, web-based engagement intervention described above. Participants were recruited between August 2013 and March 2014 in two psychiatric hospitals in the Federal State of Hessen, Germany. We included adult inpatients with a Turkish migration background and an ICD-10 F3 or F4 principal diagnosis (depressive, somatoform, anxiety, or adjustment disorder) in their first or second week of treatment. Migration background was categorized as present when one or both parents were not born in Germany (Schenk et al., 2006). Patients with bipolar disorders, acute psychosis, substance abuse disorders, neurodegenerative diseases, and a primary diagnosis of eating disorders were excluded.

During the study period, nearly all eligible patients were contacted (about 95%; see Figure 1). About 60% of the contacted patients participated in the study. Self-reported reasons for non-participation included shame, the current mental state, the duration of the study, lack of reading ability or lack of schooling, little experience in using computers, and the planned storage of study data. During the first half of the study period, participants were randomly assigned to the experimental conditions (engagement intervention or active control intervention). In the second half, groups were gender-matched to prevent a bias in the results due to an unequal gender distribution and increase internal validity of the study. Three patients dropped out shortly after the initiation of the trial. Reasons for discontinuation were exhaustion, as well as language difficulties and problems with the contents of the questions. The final total study sample comprised *N* = 26 inpatients.

We hypothesized that patients working with Sağlığa Doğru were going to feel better prepared for therapy and be more strongly motivated to engage in therapy than those working with the PMR, and that personal and treatment control beliefs as well as self-efficacy would be stronger after using the engagement intervention than before, while external-fatalistic control beliefs and threatening illness perceptions would diminish after using Sağlığa Doğru.

### Process of the Study Trial

Participants could choose their preferred language, as all instruments and both interventions were provided in German and Turkish. They completed all questionnaires and the intervention on a computer in the presence of a bilingual research assistant (D.Z.). The research assistant was ready to provide help at any time, while paying attention to ensure standardized test conditions. Written instructions were given for the individual parts of the study. The participants could take a break or discontinue assessments at any time without any consequences. To make participation less taxing, all questionnaires (see below) were completed in a morning session. In the afternoon, participants worked with the intervention, provided feasibility feedback, and completed the questionnaires for the post-assessment.

#### Measures

Clinical diagnoses were reported by the treating physician or psychologist after receiving written consent. Socio-demographics, migration-related characteristics, and dimensional psychopathology (see Supplementary Materials) were assessed at the beginning. Questionnaires about illness concept and self-efficacy (Brief IPQ, IPQ-R Personal and Treatment Control Scales, KKG External-fatalistic Control Scale, and SWE) were applied before and after the interventions. Questions regarding acceptance and feasibility were completed at the end of the interventions. All self-rating questionnaires were provided on a computer in German or Turkish according to the participants’ choice.

##### The Brief Illness Perception Questionnaire (Brief IPQ)

The Brief IPQ (nine items) assesses the cognitive and emotional representations of illness (Broadbent et al., 2006). Response options range from 0 to 10 with labeled endpoints. Item 9 (illness causes) has an open response format and was not used in this study. Sum scores range from 0 to 80, with higher scores indicating a more pessimistic and threatening illness representation. Broadbent and colleagues (2006) demonstrated its validity and reliability. Turkish and German versions of the Brief IPQ were available (Weinman et al., 2012).

##### The Revised Illness Perception Questionnaire (IPQ-R)

The IPQ-R scales ‘Personal Control’ (six items) and ‘Treatment Control’ (five items) (Moss-Morris et al., 2002) were used to assess the individual’s assumed self-efficacy and efficacy of treatments, respectively, for controlling the disorder. Response options range from 1 (‘strongly disagree’) to 5 (‘fully agree’). High values indicate high controllability of the disorder by the respective domain. Reliability and validity of the IPQ-R have been confirmed repeatedly (e.g. Moss-Morris et al., 2002). German and Turkish versions of the IPQ-R were available online (Weinman et al., 2012).

##### Locus of Control Inventory for Illness and Health (KKG)

The KKG scale ‘External-fatalistic control’ (Lohaus & Schmitt, 1989) captured the extent to which a patient is convinced that his/her complaints depend on chance, fate, or luck. Its seven items are answered from 1 (‘not at all’) to 6 (‘fully agree’); sum scores range from 7 to 42. Higher values indicate a higher conviction of external-fatalistic control of the illness. Acceptable reliability and validity has been shown (Lohaus & Schmitt, 1989). As no Turkish version was available, it was translated following the forward-backward-translation method (Brislin, 1970).

##### Generalized Self-Efficacy Scale (SWE)

Based on ten items, the SWE (Schwarzer & Jerusalem, 1995) measures an optimistic anticipation of one’s competence to cope with a situation successfully. It shows convincing evidence of validity and good psychometric properties (Luszczynska et al., 2005). Response options range from 1 (‘not at all true’) to 4 (‘exactly true’) and sum scores range from 10 to 40. The reliability of the German (Jerusalem & Schwarzer, 1999) and Turkish version (Yeşilay et al., n.d.) was satisfactory (Luszczynska et al., 2005).

##### Acceptance and Feasibility

At the end of the interventions, patients provided their global evaluation of the interventions by rating them on a scale from 0 to 10, with higher values indicating a better rating. Subsequently, they answered to four items (‘Are you motivated to engage in therapy?’, ‘After using the tool, do you feel better prepared for therapy?’, ‘Would you recommend this tool to other patients?’, and ‘Was the tool easy to handle?’) on a rating scale ranging from 0 ‘no, not at all’ to 10 ‘yes, absolutely’. The research assistant noted whether participants used the computer and the interventions without assistance and how much time participants spent using the interventions.

#### Statistical Analyses

The distribution of continuous variables was assessed for normality using Q-Q plots. One univariate outlier was detected: one participant reported 17 years of education because of his university degree. Since all other participants had reported 2-12 years of schooling, his value was replaced with the maximum schooling duration (i.e. 12 years). Univariate normality was assessed with Shapiro-Wilk tests and confirmed for all variables except for ‘German language proficiency’, most feasibility variables (see Table 3), and self-efficacy (SWE pre and post). Homoscedasticity was inspected visually via box-plots and tested statistically with Bartlett’s test for normally distributed variables or Fligner-Killeen test for non-parametric variables. For all variables, homoscedasticity was confirmed (all *p* > .05), with the exception of treatment control pre (*p* = .049).

First, the experimental groups were compared regarding socio-demographic, clinical, and feasibility variables. Discrete variables were coded dichotomously and their distribution was checked with 2x2 cross tables. Group differences were assessed using a χ^2^ test or Fischer’s exact test in the case of cells with a count less than 5. For sample comparisons in continuous variables (see Table 2 and Table 3), *t*-tests for normally distributed variables and Mann-Whitney-Wilcoxon U tests for nonparametric variables were applied. Then, the effectivity of the engagement intervention in comparison to the active control intervention with regard to treatment-related variables was analyzed using analyses of variance (ANOVAs) for repeated measures with time (pre vs. post) as within-subjects-factor and experimental group (engagement intervention vs. active control intervention) as between-subjects-factor for each variable. Since self-efficacy (SWE) was not normally distributed, an equivalent nonparametric analysis was conducted additionally using the package nparLD in R (Noguchi et al., 2012). For the group that had worked with the engagement intervention, contrast analyses (one-sided *t*-tests for dependent samples: pre vs. post / Wilcoxon signed rank test with continuity correction) were carried out to differentiate whether the observed effects originated from an improvement through the use of the engagement intervention, and were not merely due to variations in the active control condition.

**Table 2:** Study Sample Characteristics

Variable	Active control intervention (*N* = 12)	Engagement intervention (*N* = 14)	Test statistic
Socio-demographic characteristics			
Age in years	36-59, 48.6 (7.3)	38-58, 47.8 (5.5)	*t*(20) = -0.31, *p* = .76
Female sex	6 (50)	7 (50)	χ^2^(1) = 0, *p* = 1
Education in years	2-11, 6.8 (2.7)	4-12, 7.4 (2.7)	*t*(23) = 0.71, *p* = .48
Being employed^a^	6 (50)	10 (71.4)	*OR* = 2.4 [0.4;17.2], *p* = .42
Migration-related characteristics			
Years since immigration^b^	9-40, 30.8 (9.6)	17-43, 28.1 (7.4)	*t*(16) = -0.75, *p* = .46
German language proficiency^c^	1-4, 3.1 (0.9)	2-5, 3.4 (0.8)	*U* = 98.5, *p* = .43
Clinical characteristics (categorical)			
Depressive disorder	9 (75.0)	11 (78.6)	*OR* = 1.2 [0.1;11.4], *p* = 1
Somatoform disorder	2 (16.7)	2 (14.3)	*OR* = 0.8 [0.1;13.4], *p* = 1
Stress or adjustment disorder	1 (8.3)	1 (7.1)	*OR* = 0.9 [0.1;72.3], *p* = 1
Comorbid disorders	10 (83.3)	8 (57.1)	*OR* = 0.3 [0.02;2.2], *p* = .22

*Note.* For continuous variables, minimum to maximum, mean and standard deviation are given. For discrete variables, the frequency and percentage rates are given.

^a^Working part-time or full-time. ^b^*n* = 2 participants in the active control group were born in Germany and are not included here. ^c^Self-reported German language proficiency (1 = very good, 5 = none).

Effect sizes and 95% confidence intervals (as far as available) are reported for all feasibility variables and treatment-related measures. For normally distributed variables, Cohen’s *d* was calculated; a value of .2 was considered a small effect, .5 a medium effect, and .8 a large effect. Cliff’s *d* was used for non-parametric continuous variables. Cliff’s *d* ranges between -1 and 1, with 0 indicating no effect; |*d*| < 0.147 was considered a negligible effect, |*d*| < 0.33 small, |*d*| < 0.474 medium, and otherwise a large effect. Generalized eta squared ($\eta_{G}^{2}$) was given as a measure of effect size for the ANOVAs described above; an $\eta_{G}^{2}$ of .02 was considered a small effect, .13 a medium effect, and one of .26 as large. *Phi* was calculated as a measure of effect size for discrete feasibility variables. A value of *phi* = .1 was considered a small effect, .3 a medium effect, and .5 a large effect.

The significance level was set at α = .05; a *p*-value < .10 was considered a statistical trend and also reported in the results section. With respect to ANOVAs with repeated measures, only statistically significant effects were reported in the results section; all *F*- and *p*-values can be obtained as Supplementary Materials. Statistical analyses were conducted using R version 3.5.0 (R Development Core Team, 2008).

## Results

### Participants

The sample consisted of *N* = 26 Turkish immigrant inpatients (see Table 2). The mean age was 48 ± 6 years, and 50% of the participants were female. On average, participants had received 7 ± 3 years of schooling and approximately 60% were employed in a part-time or full-time job. Self-reported German language proficiency was moderate, even though 29 ± 8 years had passed since immigration and two participants were born in Germany. The most frequent main diagnosis was depression (77%), followed by somatoform disorder (15%), and stress or adjustment disorder (8%). About 70% of participants had one or more comorbid diagnoses. There were no statistically significant differences between the experimental groups in terms of socio-demographic and clinical characteristics.

### Acceptance and Feasibility

The overall rating for Sağlığa Doğru was better than that for the PMR and participants working with Sağlığa Doğru felt better prepared for therapy (see Table 3). Participants in both groups showed statistically similar levels of motivation to engage in therapy and willingness to recommend their tool to other patients.

**Table 3:** Acceptance and Feasibility of the Interventions

Variable	Active control intervention^b^	Engagement intervention^c^	Test statistic	Effect size [95% CI]
Overall rating	5.3 (2.5)	8.4 (1.6)	*t*(18) = 3.63, *p =* .002	Cohen’s *d* = 1.48 [0.55; 2.41]
*‘Are you motivated to engage in therapy?’*	8.1 (2.5)	8.7 (1.7)	*U* = 86.5, *p* = .65	Cliff’s *d* = .11 [-.34; .51]
*‘After using the tool, do you feel better prepared for therapy?’*	3.6 (3.1)	7.0 (2.6)	*U* = 115, *p* = .013	Cliff’s *d* = .60 [.10; .86]
*‘Would you recommend this tool to other patients?’*	7.2 (2.8)	7.9 (2.1)	*U* = 94, *p* = .62	Cliff’s *d* = .12 [-.33; .53]
*‘Was the tool easy to handle?^a^*	8.0 (2.7)	9.4 (1.1)	*U* = 21.5, *p* = .50	Cliff’s *d* = .23 [-.45; .74]
Use of the intervention without assistance *[N (%)]*	5 (45.5)	7 (50)	χ^2^(1) = 0.0009, *p* = .98	*phi* = 0.08
Time working with the intervention *(minutes)*	31.7 (6.8)	49.6 (6.9)	*t*(23) = 6.34, *p <* .001	Cohen’s *d* = 2.61 [1.51; 3.71]

*Note.* Unless otherwise indicated, *M* (*SD*) are presented. Rating scales ranged from 0 ‘no, not at all’ to 10 ‘yes, absolutely’.

^a^Only participants that used the intervention without assistance. ^b^*N* = 12. ^c^*N* = 14.

Only half of the participants were able to use the interventions without assistance, regardless of the experimental condition. However, those who used the interventions by themselves indicated that they were very easy to handle. Participants worked approximately 28 minutes longer with Sağlığa Doğru than with the PMR.

### Illness Perception and Self-Efficacy

Brief IPQ threatening illness perceptions decreased on a descriptive level after using Sağlığa Doğru as expected, while there was no change in the PMR-condition. The contrast analysis confirmed a statistical trend in the expected direction (Cohen’s *d* = -0.43, see Table 4). After using Sağlığa Doğru, beliefs in personal (Cohen’s *d* = 0.34) and treatment control (Cohen’s *d* = 0.20) increased, and beliefs in external-fatalistic control decreased significantly (Cohen’s *d* = -0.60). Self-efficacy increased after working with Sağlığa Doğru, while it decreased after working with the PMR with a small and statistically significant effect for the group*time interaction ($\eta_{G}^{2}$ = 0.024) that was confirmed by the nonparametric approach (Wald-type and ANOVA-type test statistic = 7.432, *df* = 1, *p* = .006). The contrast analyses confirmed a small effect and a statistically significant increase in self-efficacy after using Sağlığa Doğru (Cliff’s *d* = 0.22).

**Table 4:** Usefulness of the Engagement Intervention Regarding Treatment-Related Variables

Variable	Active control intervention^a^		Engagement intervention^b^		ANOVA (group*time interaction)^c^		Contrast analyses^d^	
	Pre	Post	Pre	Post	Test statistic	$\eta_{G}^{2}$	Test statistic	*d* [95% CI]^e^
Illness concept (Brief-IPQ)	58.7 (8.5)	58.6 (7.6)	60.3 (6.0)	57.0 (7.5)	*F*_(1, 24)_ = 1.18, *p* = .288	0.012	*t*_(13)_ = 1.62, *p* = .065	-0.43 [-1.22; 0.35]
Personal control (IPQ-R)	17.3 (2.1)	16.7 (3.9)	17.4 (3.9)	18.8 (4.1)	*F*_(1, 24)_ = 2.27, *p* = .145	0.020	*t*_(13)_ = -1.36, *p* = .111	0.34 [-0.44; 1.13]
Treatment control (IPQ-R)	14.7 (2.8)	14.9 (3.6)	15.8 (5.1)	16.7 (4.9)	*F*_(1, 24)_ = 0.17, *p* = .683	0.001	*t*_(13)_ = -0.74, *p* = .236	0.20 [-0.58; 0.98]
External-fatalistic control (KKG)	17.0 (6.3)	18.8 (7.1)	16.8 (6.4)	15.0 (6.9)	*F*_(1, 24)_ = 3.94, *p* = .059	0.019	*t*_(13)_ = 2.26, *p* = .021	-0.60 [-1.40; 0.19]
Self-efficacy (SWE)	15.7 (4.1)	14.6 (3.8)	17.4 (6.4)	19.9 (8.2)	*F*_(1, 24_*_)_* = 6.81, *p* = .015	0.024	*V* = 15.5, *p* = .034	0.22 [-0.24; 0.60]

*Note. M* (*SD*) are presented.

^a^*N* = 12. ^b^*N* = 14. ^c^All main effects for group and time were statistically not significant in ANOVA and are not shown. ^d^Pre-post comparison for the engagement intervention group only (see Statistics section). ^e^Cohen’s *d* for normally distributed data, Cliff’s *d* for SWE (not normally distributed).

## Discussion

Our work aimed at developing and piloting a culture-tailored intervention assisting Turkish immigrant inpatients to engage in psychotherapeutic treatment. In a proof-of-concept study, this intervention was rated better than an active control intervention, in particular concerning a better preparedness for psychotherapy. Self-efficacy and personal and treatment control beliefs improved through working with Sağlığa Doğru, while threatening illness perceptions and external-fatalistic control beliefs diminished.

Multicultural, web-based MI interventions have received positive feedback before, particularly regarding less shame, embarrassment, and discomfort compared to face-to-face group interventions (Osilla et al., 2012). Our study demonstrated that a web-based intervention is applicable even in a group of relatively low-educated immigrants, but the pilot trial showed that half of the sample was unable to use the computer and the web-based interventions on their own. We assume that the recruitment strategy of the current study (i.e., approaching potential participants in-person during specialized inpatient treatment for Turkish migrants) resulted in a sample that was potentially older and less digitally literate than participants who are typically included into randomized controlled trials, particularly into trials on web-based and app-based interventions with inclusion criteria such as having access to the internet (e.g., Heim et al., 2020). Understanding this barrier to implementation could be addressed by an even more rigorous emphasis on user-centered design for the target population (Burchert et al., 2019) or through task-sharing with Turkish-speaking non-therapists (e.g. nursing staff) assisting patients with low technical or digital literacy (Rebello et al., 2014).

The improvements in self-efficacy and personal control beliefs indicate the engagement intervention’s capability to strengthen the belief in one’s own coping abilities. The beliefs that health depends on chance, fate, or luck diminished after working with Sağlığa Doğru. However, even though the illness perception was *less* threatening, it remained in the range of a rather pessimistic and threatening concept of disease. It has been shown previously that a threatening illness perception was associated with poor psychological health and low motivation for psychotherapy (Petrie & Weinman, 2012). While this highlights the relevance of Sağlığa Doğru, it also suggests that continuous work is needed to achieve longer lasting changes in illness perception (Petrie et al., 2012).

### Limitations

This proof-of-concept study comprised a small sample, limiting the generalizability of the present findings. Only 60% of patients were willing to participate in the study, implying that participant burden due to study duration and concerns about data storage were relevant barriers towards participation. German and Turkish language versions of questionnaires and interventions were provided to the participants ad libitum, including the options to switch between language versions and use both versions. This approach was well received and facilitated participation but tracking of the use of language versions was not possible within the system and hence, no further analysis could be undertaken regarding language use. For most Turkish-language versions of the questionnaires, psychometric properties, cultural validity, and measurement equivalence with the German versions have not been established to a satisfactory degree, which might compromise reliability and validity of the findings regarding treatment-related variables. Contrast analyses were carried out to give a first impression of the effect of the engagement intervention on treatment-related variables, but effects need to be replicated in larger trials since statistical power was, at best, acceptable due to the small sample size. Wide confidence intervals containing zero point out that the estimates are imprecise and cannot be readily transferred to a population level. The intervention material included no female sample patient. Therefore, the identification with the (male) sample patient might have differed between male and female participants.

### Conclusions

The present proof-of-concept study gave an example of how to adapt psychoeducational information and foster treatment engagement in Turkish immigrant inpatients in a one-session, web-based intervention. While we found promising first results, the effect of the engagement intervention on actual treatment engagement and treatment outcome is still to be evaluated. Further evaluation is also needed regarding whether a one-session intervention is sufficient, or whether more sessions are necessary to create a reliable effect regarding treatment engagement. The evident limitations notwithstanding, this study provided a novel approach to fostering the engagement of an immigrant population in psychotherapy. It might encourage the further development and application of culturally tailored, web-based treatment elements which facilitate the delivery of psychotherapy or single techniques (e.g., PMR as a relaxation technique). Treatment enhancement by web-based interventions can add language and cultural resources in a scalable way and bridge gaps in the field of immigrant and minority psychotherapy. Clinical applications may be realized for immigrant and minority patients undergoing professional treatment to increase readiness for and thereby effectiveness of psychotherapy. Further applications can be envisioned to facilitate the uptake of professional treatment by using culturally tailored, web-based interventions to bridge gaps in mental health literacy and foster openness for psychotherapy in the most vulnerable populations (e.g. asylum seekers and refugees (Böttche et al., 2021), as well as other socio-economic disadvantaged groups, or adolescents and young adults).

## Supplementary Materials

The Supplementary Materials include the Turkish and German versions of the Sağlığa Doğru intervention script, additional analyses of the sample characteristics using self-report measures for dimensional psychopathology, test statistics for main effects (Table 4), and the full dataset including a codebook (for access see Index of Supplementary Materials below).



ReichH.
ZürnD.
MewesR.
 (2021a). Supplementary materials to "Engaging Turkish immigrants in psychotherapy: Development and proof-of-concept study of a culture-tailored, web-based intervention"
[Research data]. PsychOpen. 10.23668/psycharchives.5156
PMC966722736398285

ReichH.
ZürnD.
MewesR.
 (2021b). Supplementary materials to "Engaging Turkish immigrants in psychotherapy: Development and proof-of-concept study of a culture-tailored, web-based intervention"
[Additional information]. PsychOpen. 10.23668/psycharchives.5155
PMC966722736398285

## Data Availability

All data, analytic methods, and study materials are available to other researchers and can be obtained from PsychArchives as Supplementary Materials to this article (Reich, Zürn, & Mewes, 2021a, 2021b).
